# Modification of the immune response by bacteriophages alters methicillin-resistant *Staphylococcus aureus* infection

**DOI:** 10.1038/s41598-022-19922-x

**Published:** 2022-09-19

**Authors:** Tomoya Suda, Tomoko Hanawa, Mayuko Tanaka, Yasunori Tanji, Kazuhiko Miyanaga, Sanae Hasegawa-Ishii, Ken Shirato, Takako Kizaki, Takeaki Matsuda

**Affiliations:** 1 Department of General Medicine, Kyorin University School of Medicine, 6-20-2, Shinkawa, Mitaka, Tokyo, 181-8611 Japan; 2 Department of Infectious Diseases, Kyorin University School of Medicine, 6-20-2, Shinkawa, Mitaka, Tokyo, 181-8611 Japan; 3 School of Life Science and Technology, Tokyo Institute of Technology, 4259 J3-8 Nagatsuta-cho, Midori-ku, Yokohama, Kanagawa 226-8501 Japan; 4 Pathology Research Team, Faculty of Health Sciences, Kyorin University, 5-4-1 Shimorenjaku, Mitaka, Tokyo, 181-8612 Japan; 5 Department of Molecular Predictive Medicine and Sport Science, Kyorin University School of Medicine, 6-20-2 Shinkawa, Mitaka, Tokyo, 181-8611 Japan; 6 Department of Traumatology and Critical Care Medicine, Kyorin University School of Medicine, 6-20-2, Shinkawa, Mitaka, Tokyo, 181-8611 Japan; 7 Present Address: Division of Bacteriology, Department of Infection and Immunity, School of Medicine, Jichi Medical University, Shimotsuke-shi, Tochigi, 329-0498 Japan

**Keywords:** Bacteriophages, Infection, Bacterial host response, Bacterial infection

## Abstract

There is an urgent need to develop phage therapies for multidrug-resistant bacterial infections. However, although bacteria have been shown to be susceptible to phage therapy, phage therapy is not sufficient in some cases. PhiMR003 is a methicillin-resistant *Staphylococcus aureus* phage previously isolated from sewage influent, and it has demonstrated high lytic activity and a broad host range to MRSA clinical isolates in vitro. To investigate the potential of phiMR003 for the treatment of MRSA infection, the effects of phiMR003 on immune responses in vivo were analysed using phiMR003-susceptible MRSA strains in a mouse wound infection model. Additionally, we assessed whether phiMR003 could affect the immune response to infection with a nonsusceptible MRSA strain. Interestingly, wounds infected with both susceptible and nonsusceptible MRSA strains treated with phiMR003 demonstrated decreased bacterial load, reduced inflammation and accelerated wound closure. Moreover, the infiltration of inflammatory cells in infected tissue was altered by phiMR003. While the effects of phiMR003 on inflammation and bacterial load disappeared with heat inactivation of phiMR003. Transcripts of proinflammatory cytokines induced by lipopolysaccharide were reduced in mouse peritoneal macrophages. These results show that the immune modulation occurring as a response to the phage itself improves the clinical outcomes of phage therapy.

## Introduction

Multidrug-resistant (MDR) bacteria threaten health worldwide. During the COVID-19 outbreak, MDR infections spread rapidly and more severely in hospitalized patients due to the longer duration of antimicrobial therapy to manage respiratory complications^1–6^. The development of alternative treatments is an urgent issue owing to the increasing number of patients with challenging infectious strains. Phage therapy utilizing bacteriophages has more than one hundred years of history^7^, and a large volume of past clinical data have demonstrated its safety as well as effectiveness to infection of bacteria developed resistance due to different mechanisms of action compared to antibiotics^8,9^. While phage therapy is being rapidly developed to treat MDR bacterial infections and successes are accumulating^10,11^, the approaches and clinical situations in which phage therapy has been applied greatly vary, and a standardized therapy protocol has not been decided upon despite the many valuable clinical trials that have been conducted in recent years. More evidence-based and well-designed clinical trials of phage therapies are needed to make phage therapy more practical^12^.

Phages themselves are not pathogens but are recognized as antigens by the human immune system, generating an immune response. Phage therapy is generally based on the bacteriolytic activity of bacteriophages; therefore, in clinical and animal studies, a phage is selected that is specific and virulent to the particular bacteria causing the infection^13,14^. However, while bacteriolysis is an essential factor for phage efficacy, the mechanisms of phage activity on the infection in vivo*,* especially via the immune system, have not been clarified well thus far. Some case studies have reported that phages successfully suppressed increased inflammatory cytokine levels due to bacterial infections^15–17^. Additionally, it has been suggested that the suppression of inflammation occurs not only as a result of a decrease in bacterial load but through an anti-inflammatory effect of the phage itself^18,19^. Although a variety of direct and indirect changes occur in bacterial infections, the immunological response of the host human to phages might influence the effects of phages. Phage therapy might show multifaceted effects, unlike treatment with antimicrobial agents, which have a well-defined point of action, since phage therapy uses microorganisms with complex structures in treatment^20^.

Methicillin-resistant *Staphylococcus aureus* (MRSA) is a representative multidrug-resistant bacterium and causative agent of skin and soft tissue infections as well as pneumonia or sepsis. PhiMR003 was previously isolated from sewage influent and demonstrates a broad host range in MRSA clinical isolates^21^. To investigate possible clinical use, we investigated the efficacy of phiMR003 in mouse wound infections with phiMR003-sensitive MRSA strains. Furthermore, we also considered whether phiMR003 could affect the host immune reaction to bacterial infection with an MRSA strain not susceptible to phiMR003.

## Results

### Efficacy of phiMR003 on wound infections with susceptible MRSA strains

**Figure 1:**
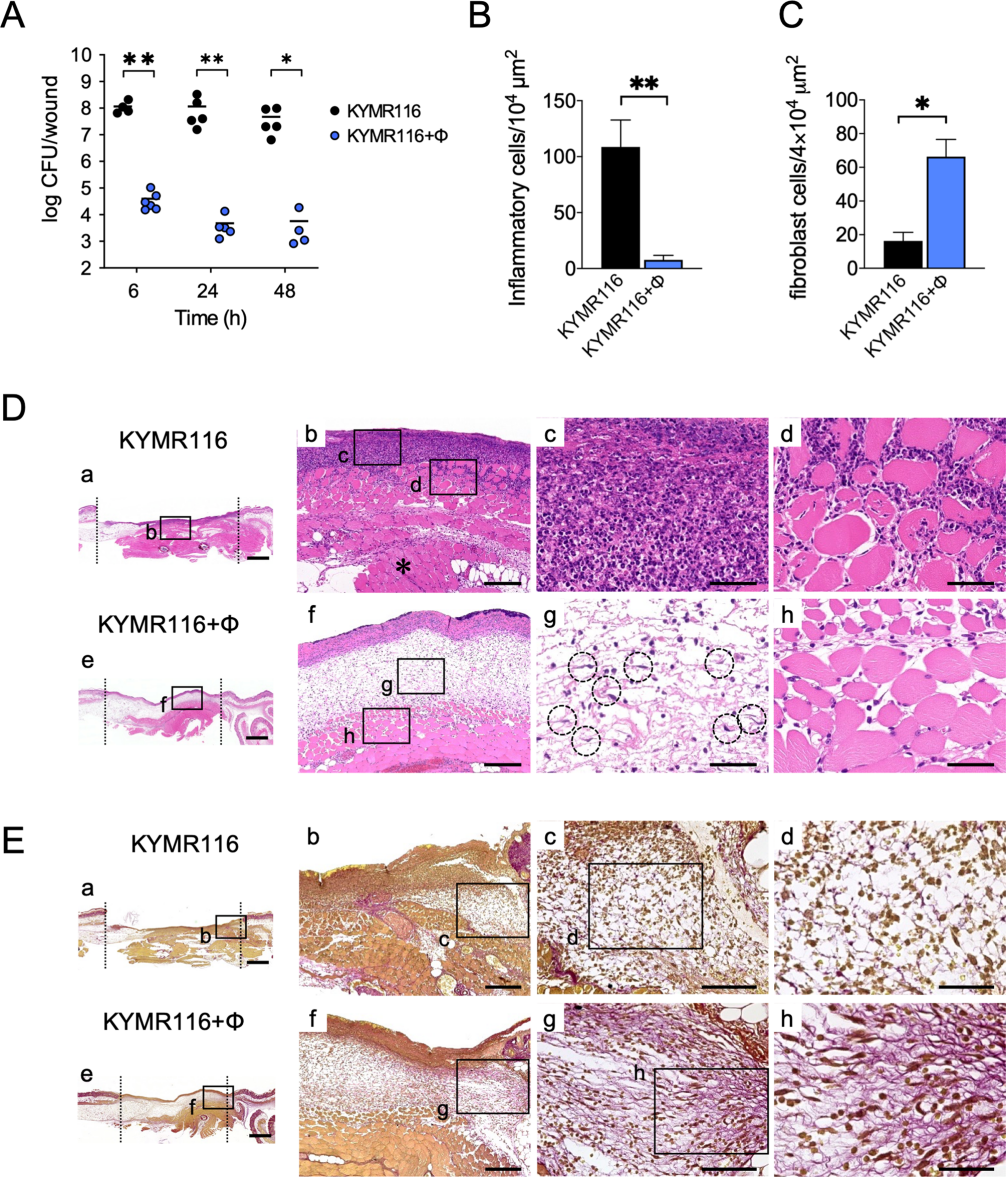
The bacterial loads and histopathology of tissues during infection with KYMR116. (**A**) The number of bacteria in the tissue was quantified at 6, 24, and 48 h after infection with KYM116. The results represent the mean CFU/wound (n = 5). (**B**) The density of the infiltrating inflammatory cells was quantified using three areas randomly chosen in the abscesses. The data indicate the means of the number of cells per 10^4^ µm^2^ (n = 3). (**C**) Emerged fibroblasts were quantified using three areas randomly chosen in the abscesses. The data indicate the means of the number of cells per 4 × 10^4^ µm^2^ (n = 3). (**D**) Mouse skin tissues observed by haematoxylin and eosin (H&E) staining at 48 h after infection. Data are representative of three mice in each group. Cells enclosed in dotted lines are fibroblasts. Scale bars, 1 mm in a and e, 200 µm in b and f, and 50 µm in c, d, g and h. (**E**) Collagen and elastin fibres in the mouse skin were detected by Elastica van Gieson staining. The micrographs of a-h in (**D**) and (**E**) are the areas indicated by squares a-h, respectively. An asterisk in (**D**)-b indicates the lesion of infection in the deep tissue. ‘KYMR116 + ϕ’ are the samples prepared from mice coinoculated with phiMR003 and KYMR116. Error bars indicate standard errors of the means (SEMs). *, *P* < 0.05; **, *P* < 0.01. Scale bars, 1 mm in a and e, 200 µm in b and f, 100 µm in c and g, and 50 µm in d and h.

**Figure 2:**
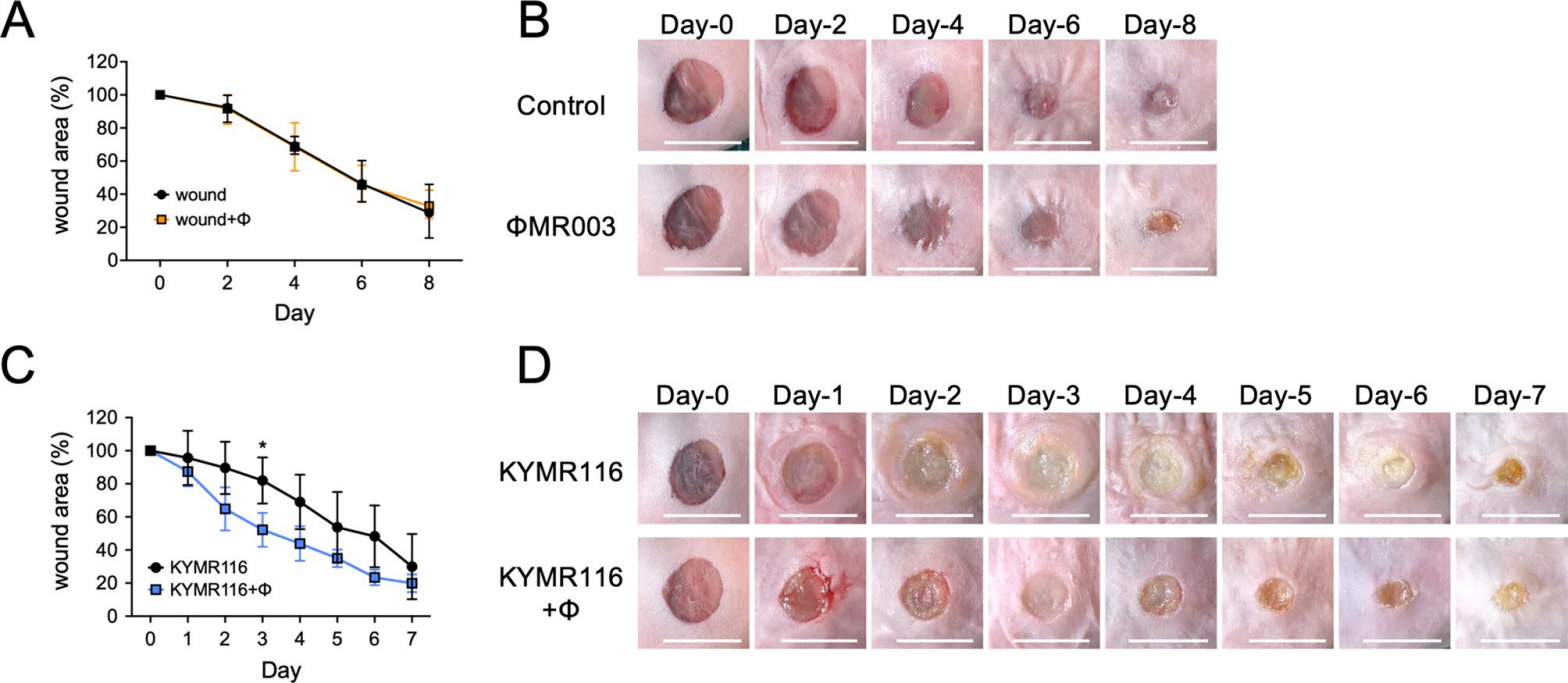
The wound closure rate of the 6.5 mm diameter skin excision. After the administration of buffer (wound) or phiMR003 (wound + ϕ), wound size was measured every other day (**A**). The effect of phiMR003 on wound closure was assayed in KYMR116-infected wounds after the administration of buffer (KYMR116) or phiMR003 (KYMR116 + ϕ) (**B**). Data indicate the mean rate of five mice in each group. Error bars indicate the standard errors of the means (SEMs). The Mann‒Whitney U test determined the significance of the data on the 3rd day (* *P* < 0.05). Two-way analysis of variance (ANOVA) was used to determine the level of statistically significant phiMR003 administration (*P* < 0.05). Representative photographs of the wound are shown in (**B**) and (**D**). Scale bars, 10 mm.

KYMR116 is a MRSA clinical isolate that is sensitive to phiMR003 and is lysed efficiently in vitro (Fig. S1). After wound site inoculation of this strain, similar numbers of viable bacterial cells and inoculated bacteria were detected until 48 h (Fig. 1A). On the other hand, the bacterial load at the wound site was strikingly decreased at 6 h following phiMR003 administration and further decreased at 24 h (Fig. 1A) (*P* = 0.0047, 6 h vs. 24 h). A considerable number of phiMR003 particles (1.0 × 10^6^ ± 1.3 × 10^6^ pfu/wound) were detected in the phage-treated tissue at 48 h. These results suggested that the viable bacterial number decreased due to the lysis of bacterial cells by phiMR003 in vivo. According to the histopathological analysis of tissue infected with KYMR116, the infiltration of numerous inflammatory cells of all morphologies with a predominance of polymorphonuclear leukocytes was observed, and these cells infiltrated between damaged muscle cells (Fig. 1B and D, see Supplementary Fig. S2). In contrast, phiMR003 administration markedly decreased inflammatory cell infiltration and significantly increased fibroblast and extracellular matrix formation (Fig. 1B–E). A significant difference in the closures of the control (SM buffer) and phiMR003-treated wounds was observed only on the 3rd day (Fig. 2C). Furthermore, two-way analysis of variance (ANOVA) was used to determine the level of statistically significant phiMR003 administration (*P* < 0.05). Additionally, abscess formation was apparently suppressed by phiMR003, and wound closure was accelerated (Fig. 2).

**Figure 3:**
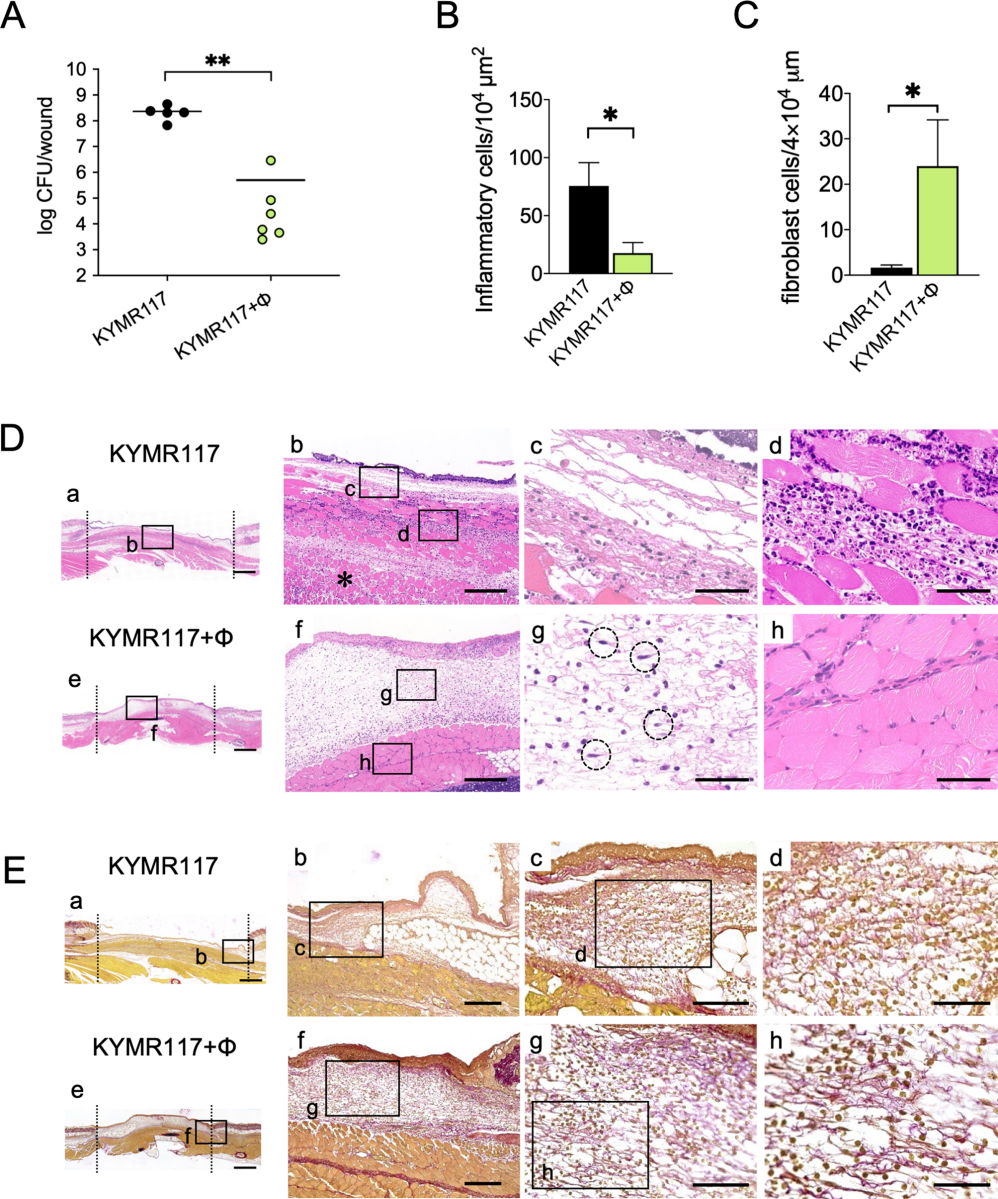
Effects of phiMR003 on the bacterial loads and histopathological analyses in KYMR117 infection. ‘KYMR117** + **ϕ in the graphs indicates the samples prepared from mice coinoculated with KYMR117 and phiMR003. (**A**) The number of bacteria in the tissue was counted 48 h after infection. The results represent the mean CFU/wound (n = 5). (**B**) The density of the infiltrated inflammatory cells was quantified using three areas randomly chosen in the abscesses. The data indicate the mean number of cells per 10^4^ µm^2^ (n = 3). (**C**) Emerged fibroblasts were quantified using three areas randomly chosen in the abscesses. The data indicate the means of the number of cells per 4 × 10^4^ µm^2^ (n = 3). (**D**) Mouse skin tissues observed by haematoxylin and eosin (H&E) staining at 48 h after infection. Data are representative of three mice in each group. Scale bars, 1 mm in a and e, 200 µm in b and f, and 50 µm in c, d, g and h. (**E**) Collagen and elastin fibres in the mouse skin were detected by Elastica van Gieson staining. Scale bars, 1 mm in a and e, 200 µm in b and f, 100 µm in c and g, and 50 µm in d and h. The micrographs of a-h in (**D**) and (**E**) are the areas indicated with squares a-h, respectively. The asterisk in (**D**)-b indicates the lesion of infection in the deep tissue. Error bars indicate the standard errors of the means (SEMs). *, *P* < 0.05; **, *P* < 0.01.

KYMR117, a highly virulent USA300 clone, is another strain that is susceptible to phiMR003 (Fig. S1). In KYMR117-infected tissue, cell necrosis was observed, and although a few inflammatory cells were observed in surface lesions, the high invasiveness of KYMR117 was revealed by inflammatory cells infiltrating deep layers and muscle cell damage (Fig. 3D-b), which were slightly observed in KYMR116-infected tissue (Fig. 1D-b). Similar to what was observed with KYMR116 infection, phiMR003 notably reduced the bacterial load of KYMR117, significantly increased fibroblasts and induced the production of extracellular matrix formation in the tissue (Fig. 3).

**Figure 4:**
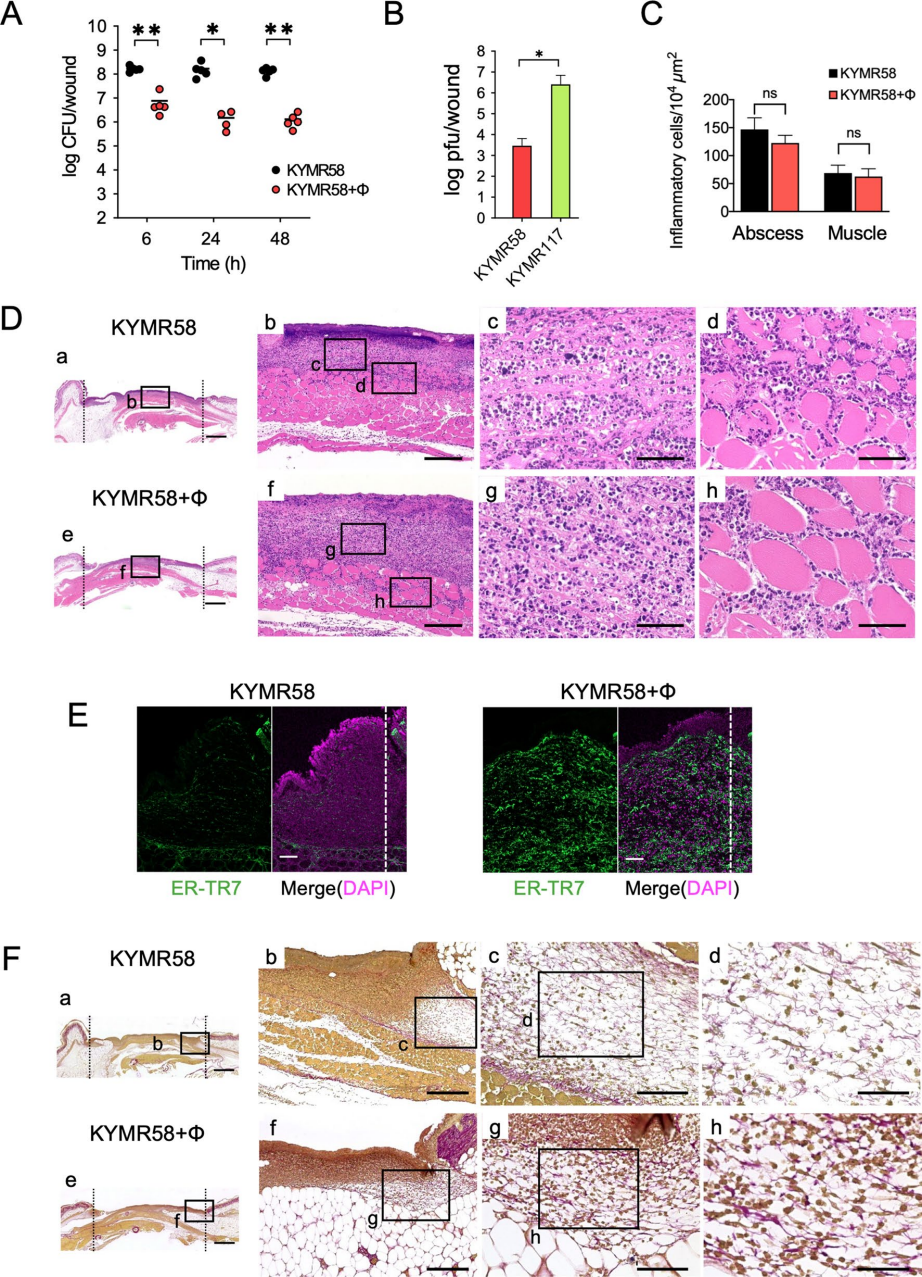
Bacterial loads and histopathological analysis of tissues infected with KYMR58; alone (KYMR58) and with phiRM003 (KYMR58 + ϕ). (**A**) The number of bacteria in the tissue was quantified at 6, 24 and 48 h after infection with KYMR58. The results shown are the mean CFU/wound (n = 4 or 5). (**B**) Phage titre in the tissue infected with KYMR58 and KYMR117 at 48 h after infection. (**C**) The density of the infiltrated inflammatory cells was quantified using three areas randomly chosen in the abscesses. The data show the mean number of cells per 10^4^ µm^2^ (n = 3). (**D**) Mouse skin tissues observed by haematoxylin and eosin (H&E) staining at 48 h after infection. Data are representative of three mice in each group. Scale bars, 1 mm in a and e, 200 µm in b and f, and 50 µm in c, d, g and h. (**E**) Immunofluorescent micrographs detecting fibroblasts (green) with anti-ER-TR7 antibody. Nuclei were stained with DAPI (magenta). Scale bars, 100 µm. (**F**) Collagen and elastin fibres in the mouse skin were detected by Elastica van Gieson staining. Scale bars, 1 mm in a and e, 200 µm in b and f, 100 µm in c and g, and 50 µm in d and h. The micrographs of a-h in (**D**) and (**F**) are the areas indicated with squares a-h, respectively. Error bars indicate standard errors of the means (SEMs). *, *P* < 0.05; **, *P* < 0.01.

**Figure 5:**
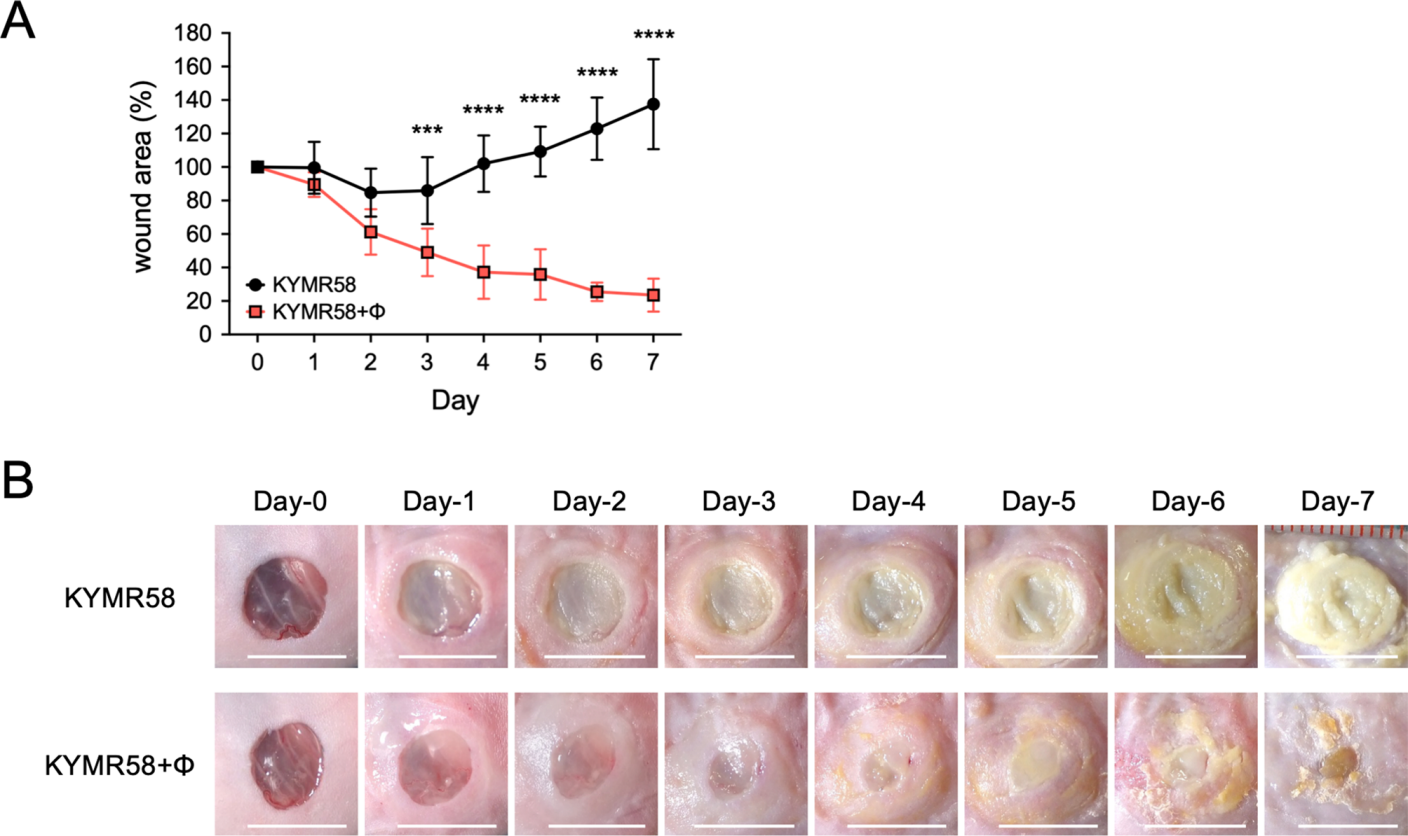
The wound closure rate of the skin excisions during KYMR58 infection. (**A**) The wound sizes were measured every day after the administration of SM buffer (KYMR58) or phiMR003 (KYMR58 + ϕ). The wound closure rate is the mean of five mice in each group. Error bars indicate the standard errors of the means (SEMs). The Mann‒Whitney U test determined the significance of the data (***, *P* < 0.001; ****, *P* < 0.0001). Representative photographs of the wound are shown in (**B**). Scale bars, 10 mm.

We next assessed whether phiMR003 has other effects in addition to its bacteriolytic activity by analysing infection with KYMR58, the only MRSA strain that phiMR003 does not lyse at all on the soft agar plates and liquid culture at high concentrations in vitro so far (Fig. S1). Similar to KYMR116 infection, an equivalent viable bacterial cell number to the inoculum was detected at 6 h and later until 48 h after infection with KYMR58 (Fig. 4A). Intriguingly, the bacterial load was slightly but significantly decreased by phiRM003 (Fig. 4A). A number of phiMR003 comparable to that in the KYMR116-infected tissue was detected in the KYMR117-infected tissue at 48 h after administration of phages; however, significantly fewer phages were detected in the KYMR58-infected tissue than in KYMR-117-infected tissue (Fig. 4B). Histopathological analysis of KYMR58-infected tissue indicated that phiMR003 did not alter the extent of inflammatory cell infiltration (Fig. 4C and D); however, fibroblast and extracellular matrix formation increased (Fig. 4E and F), and there was a significant decrease in wound size (Fig. 5).

### Effect of phiMR003 on cytokine levels in MRSA infection

Interleukin (IL)-1β is a potent cytokine promoting neutrophil migration and triggering abscess formation in response to *S. aureus* skin infection. The treatment of KYMR116-infected tissue with phiMR003 resulted in a highly significant reduction in the inflammatory cell infiltrate. Therefore, we assessed the effect of phiMR003 on the production of IL-1β together with two other proinflammatory cytokines: IL-6 and tumour necrosis factor (TNF)-α.

**Figure 6:**
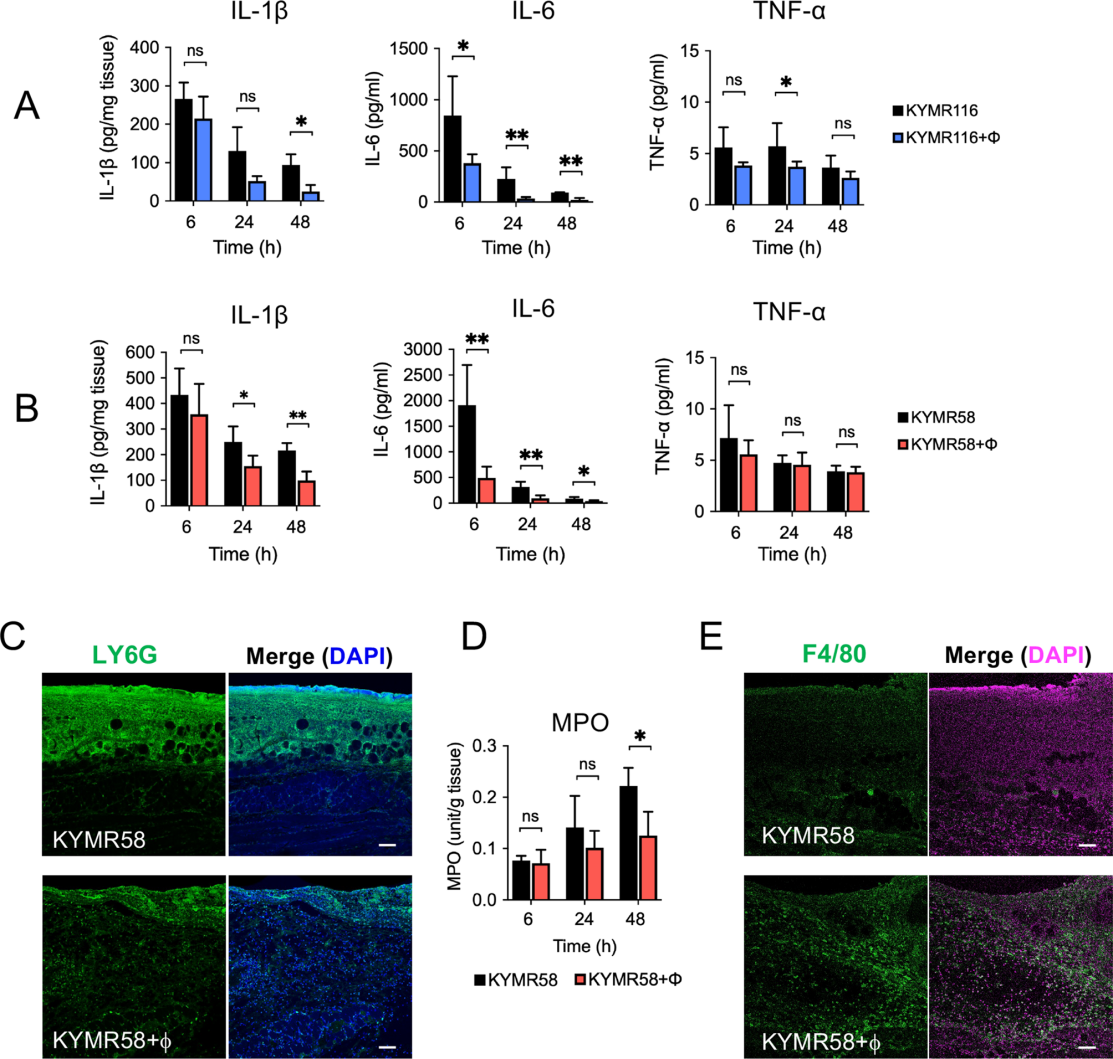
Effects of phiMR003 on the inflammatory responses induced by MRSA. Samples were prepared at 24 h after infection with KYMR116 or KYMR58 followed by the administration of phiMR003 (KYMR116 +ϕ or KYMR58 + ϕ) or SM buffer (KYMR116 or KYMR58) to the mice. IL-1β, IL-6, and TNF-α in the samples were quantified by ELISA (n = 5) after infection with KYMR116 (**A**) and KYMR58 (**B**). Error bars indicate the standard errors of the means (SEMs). *, *P* < 0.05; **, *P* < 0.01. Representative data from three repeated experiments are shown. The detection of neutrophils (**C**) and macrophages in mouse skin tissue (**E**) by immunofluorescent staining with anti-Ly-6G (green) and anti-F4/80 (green) antibodies. Nuclei are stained with DAPI (blue or magenta). Neutrophils in the tissues were quantified by myeloperoxidase assay (**D**). Scale bars, 100 µm.

In the present study, we measured the cytokine levels using tissues and serums without wounding and bacterial infection to evaluate the normal level of inflammatory cytokines. The IL-1β level of normal tissue was 3.2 ± 1.2 pg/mg tissue (n = 4), and the levels of IL-6 and TNF-α in the serum were less than the detection limits (less than 7.8 pg/ml and 0.8 pg/ml, respectively). The IL-1β level in KYMR116-infected wound tissue at 48 h decreased to approximately 1/3 that at 6 h (265.9 to 94.1 pg/mg tissue). This reduction rate increased to 1/9 (214.8 to 24.6 pg/mg tissue) with phiMR003 administration, and the IL-1β level was significantly lower than that in the tissue without phiMR003 treatment at 48 h (Fig. 6A). phiMR003 decreased IL-6 levels more rapidly and significantly than it decreased TNF-α levels. A small but significant decrease in TNF-α was observed in phiMR003-treated tissue at 24 h (Fig. 6A). These results suggested that the systemic immune response to surface MRSA infection is suppressed through the administration of phiMR003 at the infection site.

phiMR003 did not reduce the number of inflammatory cells in the KYMR58-infected tissue, but it did increase the number of fibroblasts and amount of extracellular matrix, and it accelerated wound closure (Figs. 4 and 5). Interestingly, phiMR003 also suppressed the IL-1β and IL-6 levels induced by KYMR58 infection (Fig. 6B). Furthermore, these impacts on cytokine levels were comparable to those observed in KYMR116 infection, despite the considerably different effects on bacterial loads.

### Effect of phiMR003 on inflammatory cells in infected tissue

*S. aureus* infections are characterized by the formation of thick-walled abscesses through the recruitment of neutrophils to skin and soft tissue infection sites. As shown in Fig. 4C and D, phiMR003 did not change the number of inflammatory cells in the infiltrate during KYMR58 infection, even though IL-1β and IL-6 levels significantly decreased. However, immunofluorescence analysis of KYMR58-infected wounds revealed that phiMR003 decreased the number of infiltrating Ly6G-positive neutrophils (Fig. 6C), which was further proven by a myeloperoxidase assay (Fig. 6D).

Macrophages have a pivotal role in bacterial infections and the determination of the immune response. Intriguingly, macrophages were abundant in tissue treated with phiRM003, as detected by F4/80 immunofluorescence staining (Fig. 6E).

### Suppression of proinflammatory cytokines in peritoneal macrophages by phiMR003

**Figure 7:**
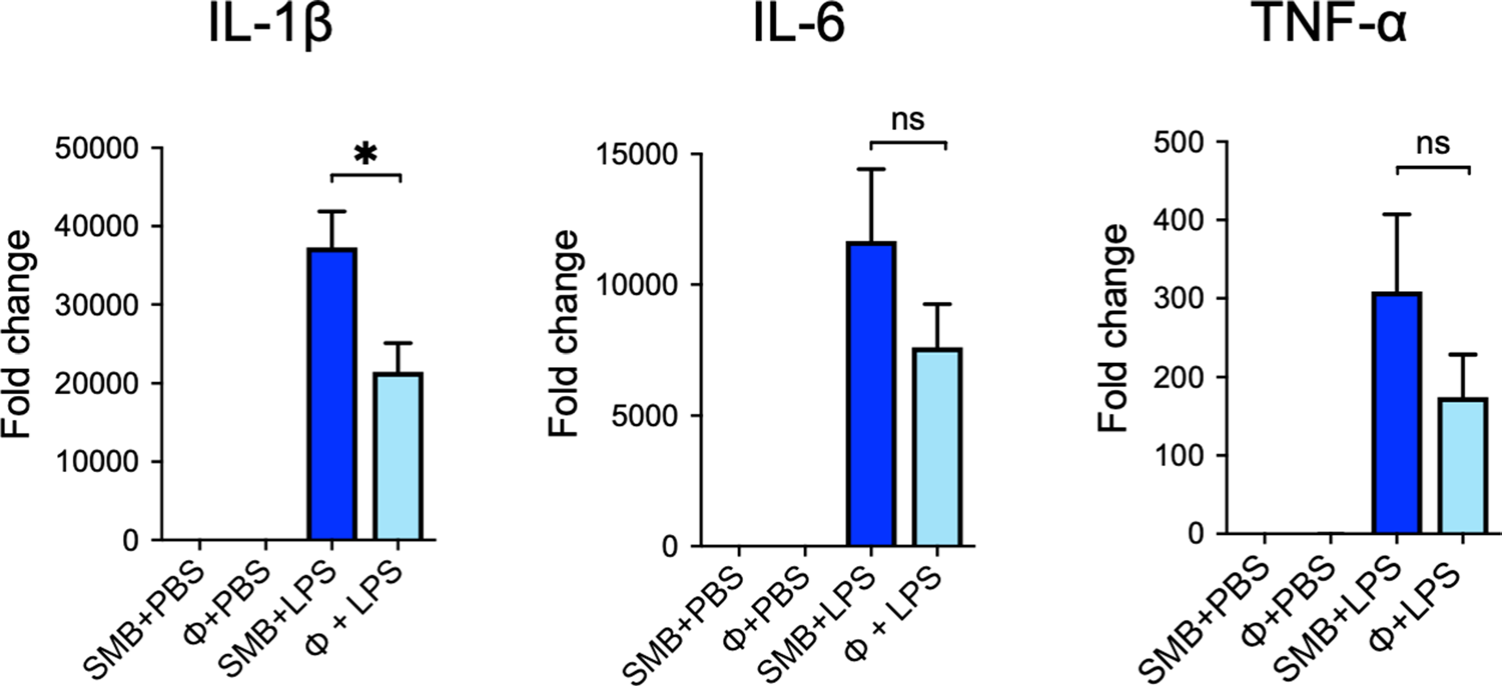
Effects of phiMR003 on the induction of cytokine transcription in mouse peritoneal macrophages. Transcripts of IL-1β, IL-6, and TNF-α were quantified in the presence (ϕ) or absence (SMB, SM buffer) of phiMR003. PBS was added instead of LPS as a control. Data indicate the mean of three independent experiments. Error bars indicate the standard errors of the means (SEMs). The Mann‒Whitney U test was used to determine the significance of the data (n.s., not significant; *, *P* < 0.05).

We investigated the effect of phiMR003 on the induction of cytokines caused by bacterial lipopolysaccharide (LPS), a potent stimulator of proinflammatory cytokines, in mouse peritoneal macrophages^22–24^. phiMR003 did not induce any change in cytokine levels without LPS; however, after induction with LPS, IL-1β transcript levels were significantly decreased (*P* = 0.025), although IL-6 and TNF-α levels were not changed (IL-6, *P* = 0.21; TNF-α, *P* = 0.22) (Fig. 7).

### Inactivation of phiMR003 results in a loss of the observed effects

**Figure 8:**
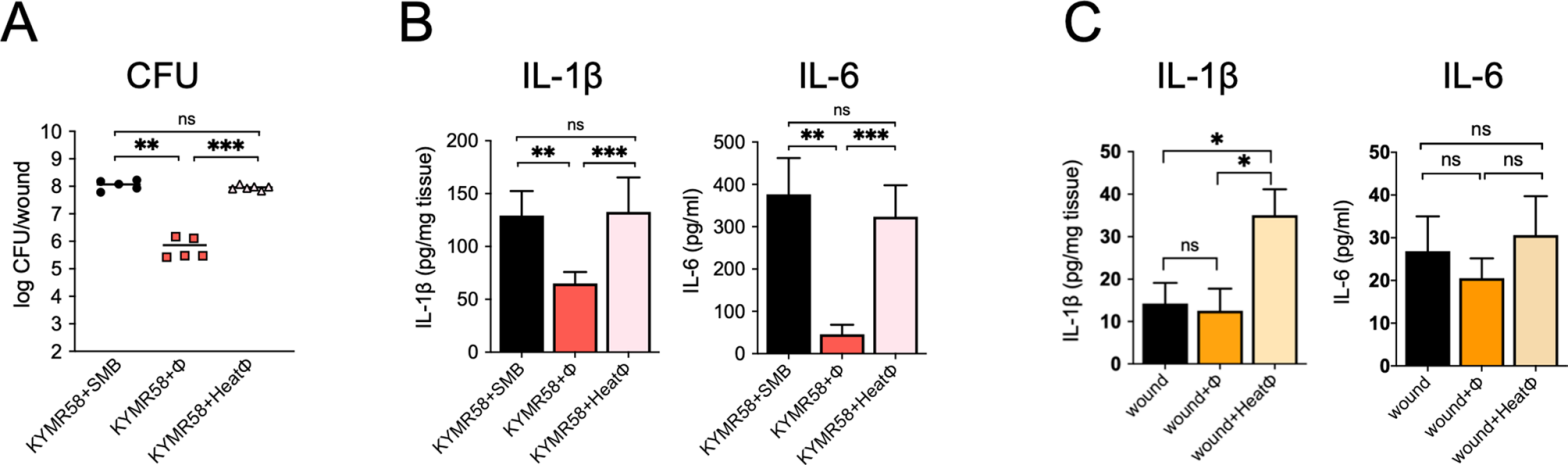
Effects of heat inactivation of phiMR003 on the bacterial load and cytokine production. (**A**) The number of bacteria in the tissue was quantified 48 h after infection. The results represent the mean CFU/wound (n = 5 or 6). (**B**) IL-1β and IL-6 were quantified using the samples prepared at 24 h after KYMR58 infection followed by the administration of mature phiMR003 (KYMR58 + ϕ), heat-inactivated phiMR003 (KYMR58 + Heatϕ) or SM buffer (KYMR58 + SMB). (**C**) Active and heat-inactivated phiMR003 were administered to the excised skin, and IL-1β and IL-6 were quantified at 24 h after infection and phage administration. Data show the mean of a number of samples (n = 5 or 6). Error bars indicate the standard errors of the means (SEMs). *, *P* < 0.05; **, *P* < 0.01.

We investigated whether the complete phage virion was required for the effects of phiMR003 on cytokine production and bacterial load in KYMR58 infection. Heat inactivation of phage virions completely suppressed the effects on the bacterial load and the levels of IL-1β and IL-6 (Fig. 8A and B). These results suggest that the recognition of intact phiMR003 virion is necessary for the response to reduce the bacterial load and suppress inflammatory cytokine production.

Wound formation itself induces inflammation through the recognition of damage-associated molecular patterns. The effects of phiMR003 on noninfected wounds were investigated, and whereas the administration of intact phiMR003 showed no induction of IL-1β or IL-6 and, heat-inactivated phiMR003 significantly induced IL-1β levels (Fig. 8C).

## Discussion

Wound healing is represented in four stages^25,26^. First, platelets, which are activated by vessel injury, initiate haemostasis and inflammation at the skin-damaged site. Then, the inflammation leads to the infiltration of immune cells to remove the damaged cells. Subsequently, inflammation is suppressed, and fibroblasts and other cells form granulation tissue. Finally, the excessive repair reaction is suppressed, and the wound heals in the tissue reconstruction phase. In the case of bacterially infected wounds, the inflammatory phase is prolonged, and the wound healing process is inhibited^25,27,28^. Infection with *S. aureus* is a well-known risk factor for chronic wound infection due to various staphylococcal toxins and enzymes delaying wound healing processes^29–32^. Neutrophils that are attracted to the wound by these virulence factors cause abscess formation, which is a typical feature of MRSA skin and soft tissue infection^33^.

In the present study, phiMR003 caused an acceleration of wound closure for wounds infected with KYMR116 and 117 by suppressing inflammation and abscess formation and increasing fibroblast and collagen formation (Figs. 1, 2, 3). Since we focused on analysing the effects of phages on the host immune system, especially the effect of phage administration on the early innate immune reaction to bacterial infection, bacteria and phages were administered almost simultaneously. In infections for which phage therapy is actually indicated, responses will be more complex because interactions between bacteria and the host immune system have already occurred, and lysed bacterial components are generated.

The efficacy of phage therapy for the treatment of infections is assumed to be due to the bacteriolytic activity of the phages in general. We observed a remarkable reduction in the bacterial loads of KYMR116 and 117 through phiMR003 administration, and comparable numbers of phages (~ 10^6^ pfu/wound) were detected in the tissue at 48 h. The number of phages was significantly higher than the number of bacteria of the KYMR58 insensitive strain (10^3^ pfu/wound) (Fig. 4B), suggesting that multiple in vivo therapies can be sufficient for the treatment of infections with susceptible strains regardless of pathogenicity. These results corroborate previous findings and support the importance of phage sensitivity assessed in vitro to the target bacterial strain^21,34^. Phages K^35^ and PYOsa^36^ have also been used for the treatment of abscess-forming infections and are candidates for *S. aureus* (and MRSA) infection treatment due to their highly bacteriolytic ability and broad host range. Since our previous study revealed that phiMR003 has a broad host range and efficiently kills clinical isolates, it is also a candidate therapeutic strain^21^.

The pathological improvement in wound infections with phiMR003-susceptible strains can be considered a result of the rapid lysis of bacteria, in line with previous studies of in vivo phage efficacies^37^. However, phiMR003 was also able to slightly reduce the bacterial load in KYMR58-infected tissue despite the strain being nonsusceptible in in vitro analyses (Fig. 4). More strikingly, abscess formation was suppressed, and wound closure was accelerated (Fig. 5). Even though the reduction in the bacterial load of KYMR58 by phiMR003 was not to the same level as that of KYMR116, phiMR003 suppressed the induced levels of IL-1β and IL-6 to similar levels (Fig. 6A and B). Furthermore, an increase in fibroblasts and extracellular matrix formation was observed in all three cases (Figs. 1, 2, 3, 4, 5). Phage treatment significantly reduced abscess size during KYMR58 infection, and its impact was greater than that during KYMR116 (susceptible MRSA) infection, suggesting that the mechanism is completely independent of bacteriolytic activities. These results led to the hypothesis that phiMR003 may act through a host cell mechanism to improve the clinical course of MRSA wound infections.

It has been implied that phages have immunomodulatory activity related to pathology in phage therapy^38^ and several previous reports have suggested that phages have a suppressive effect on inflammation^39–42^. Van Belleghem et al. investigated the effects of *S. aureus* and *P. aeruginosa* phages on gene expression and demonstrated that all five examined phages suppress the expression of cytokines and chemokines and promote the anti-inflammatory interleukin 1 receptor antagonist which also promotes several other pro-inflammatory cytokines^39^. Furthermore, phages can suppress the NF-kB signalling pathway and reduce inflammation^40–42^.

Toll-like receptor (TLR)9 recognizes unmethylated CpG motifs in the viral genome^43–45^, resulting in the induction of proinflammatory cytokines^43^ and the interferon regulatory factor (IRF) signalling pathway, which has been implicated in the induction of IL-1 receptor antagonists and suppressors of cytokine signalling (SOCS)^46–48^. Therefore, the stimulation of the immune response to the virus through recognition might lead to decreases in IL-1β, IL-6 and TNF-α in MRSA infection.

phiMR003 appears to modify the innate immune response to MRSA infection, causing a decrease in the number of neutrophils (the main IL-1β-producing cells in *S. aureus* infection^49^ and an increase in the number of macrophages observed in the inflammatory cell infiltrate (Fig. 6). LPS is a well-known stimulator of innate immunity through TLR4 and is not contained in Gram-positive bacterial cells such as *S. aureus*. At the same time, recent studies using mice deficient in each TLR suggest that TLR4 and TLR2 play an essential role in the defence against *S. aureus*^50,51^. PhiMR003 significantly suppressed LPS-stimulated production of IL-1β in peritoneal macrophages, although IL-6 and TNF-α transcripts were unchanged (Fig. 7). Further investigation of the effects on macrophages in vivo and in vitro is needed to clarify the mechanisms of proinflammatory cytokine suppression and inflammatory cell attraction by phiMR003.

Since inflammation is essential to eradicate bacteria through the promotion of phagocytosis and the degradation of the pathogen, the suppression of inflammation and decreased bacterial load are likely to be contradictory. The present study found that the excessive inflammation associated with infection could be suppressed by phiMR003, resulting in a short-term improvement in the pathological condition. However, this anti-inflammatory effect is not believed to improve the pathological condition in all cases. In previous reports, Pf phage, a *Pseudomonas* lysogenic phage, suppressed inflammation but was thought to have resulted in chronic *P. aeruginosa* skin infections in some cases due to the anti-inflammatory effects generated by the response to the virus in host cells^52,53^. This suggests that long-term suppression of inflammation by phiMR003 could worsen the infectious pathology or increase the risk of infections by other bacteria. Therefore, combination treatment with antimicrobials will be strongly recommended.

Another therapeutic effect of phage therapy may be the enhancement of bacterial clearance by host cells, which needs to be studied in more detail. TLR-9 activation by CpG-oligonucleotides is suggested to also enhance intracellular ROS production and the bactericidal activity of macrophages^54^. Phage efficacy is abolished in neutropenic mice with *P. aeruginosa* respiratory tract infections^55^. This finding suggests that the synergistic effects of phage administration and the host immune system, especially neutrophils, are essential to eradicate bacteria^56^. The effector cells or factors that contribute to decreases in bacterial load through phiMR003 administration first need to be clarified.

Heat inactivation of phiMR003 phage virion abolished the observed effects on bacterial load and IL-β and IL-6 levels (Fig. 8A and B). Additionally, a slight but significant increase in IL-1β after the administration of heat-inactivated phages was observed. These results provide insight into the fact that active phage virions are recognized differently from denatured phages and induce specific immune responses. It has been shown that the anti-inflammatory effect of Pf phage is related to TLR3, which recognizes viral double-stranded DNA^57^. The analysis of the recognition of phiMR003 phage virion following a signalling response should be explored to clarify the mechanism on host immunity.

Previous in vivo studies have shown that phage administration can modify the immune response^58–60^. It has been reported that pro- and anti-inflammatory responses are induced in peripheral blood mononuclear cells by *S. aureus* and *Pseudomonas aeruginosa* phages^39^. Intraperitoneal administration of T4 phage resulted in immunosuppression and suppressed monocyte infiltration and T-cell activation, resulting in extended survival of mouse skin allografts^57^. Four therapeutic phages, including the T4 phage, reduced the onset of mouse collagen-induced arthritis by 37.5% after intraperitoneal administration^58^. These results indicate that phages stimulate the innate and acquired immune responses and modulate the immunity induced by other stimuli, leading to the immunosuppression and anti-inflammatory effects observed in vitro experiments^59,60^. On the other hand, phiMR003 did not affect the slightly induced levels of IL-1β and IL-6 caused by wound creation alone since the damage-associated molecular patterns (DAMPs) caused by skin excision are recognized and cause the induction of the innate immune system^61^. These results are in good agreement with the reports that the effects of phage therapy alone are limited without bacterial infection, even when taking into account their known immunomodulatory effects^62^.

The results presented here indicate that phiMR003 reduces the bacterial load, suppresses inflammation, and improves wound infections, probably by modifying the host immune response to bacterial infection, as especially seen in the nonsusceptible KYMR58 strain. Notably, it remains possible that the effects on host immunity described here might be the specific reaction of phiMR003 resulting from infection with KYMR58. Further investigations of the immunological reactions of phiMR003, such as effects on infections with other bacterial species that are not the host of this phage, are required to deepen the understanding of the effects of phages on pathology in bacterial infections.

### Conclusion

The efficacy of phage therapy was thought to be due to a decrease in the number of bacteria exacerbating infections. In the present study, it was suggested that phiMR003 improved wound infections in mice by modulating the host immune response and changing the pathology of bacterial infection in addition to decreasing the bacterial burden through its bacteriolytic activity. The suppression of inflammation through the reduction in proinflammatory cytokine levels and the improvement in wound conditions through the modification of the phage-induced immune response were not related to the reduction in bacterial load.

An analysis of the immunological response after infection suggests that phage therapy will be required for more practical treatment. The findings of this study provide new perspectives for future phage therapy. Further research analysing the effects of phages on infection sites will contribute to the design of appropriate treatment plans and determine the impact of phage therapy.

## Methods

All experimental protocols were approved by the Genetic Recombination Experiment and Pathogen Handling Experiment Committee of Kyorin University (No. 205). The Animal Experiment Committee of Kyorin University (No. 224) approved all procedures including animal experiments, and the study was carried out in compliance with the ARRIVE guidelines. All methods were carried out in accordance with relevant guidelines and regulations.

### Phage and bacterial strains

The MRSA phage phiMR003 used in the present study was previously isolated from sewage influent^21^. phiMR003 was propagated using methicillin-susceptible *S. aureus* (MSSA) RN4220^21^ from a liquid culture of Luria–Bertani (LB) broth supplemented with 1 mM MgCl_2_ and 1 mM CaCl_2_ and prepared as described previously with slight modification^63^. PhiMR003 was precipitated by the addition of 10% (w/v) polyethylene glycol (PEG) (Ave. MW, 8000, P2139, Sigma‒Aldrich, St. Louis, USA) and 4% (w/v) NaCl at 4 °C after the removal of residual bacterial DNA and RNA phage lysate by treatment with 1 µg/mL DNase I (Worthington Biochemical Corp, Lakewood, USA) and RNase A (P4875, Sigma‒Aldrich, St. Louis, USA) for 1 h at 37 °C. PEG was removed by CHCl_3_ treatment, and phage particles were collected by CsCl density-gradient centrifugation. The phage was purified and concentrated by an Amicon Ultracel 100 K (Merck-Millipore, Cork, Ireland), passed through a 0.45 µm pore-sized membrane filter (Merck-Millipore, Cork, Ireland) and stored at 4 °C. Before using it for experiments, the titre was checked by the plaque formation method (PFU/mL). To prepare heat-inactivated phage, the purified phage fraction was heated at 98 °C for 20 min. No plaque formation was confirmed using *S. aureus* RN4220 and KYMR116.

The phiMR003-sensitive methicillin-resistant *S. aureus* (MRSA) strains MR116 and MR117 (renamed KYMR116 and KYMR117, respectively) and the methicillin-insensitive strain MR58 (renamed KYMR58) were previously isolated from patients^21,64^. In addition, KYMR117, isolated from a patient with diabetes, was identified as a *pvl*-positive USA300 clone^64^.

The MRSA strains were routinely cultured with LB broth or LB agar plates at 37 °C. For mouse infection, bacterial cells growing in TSB medium (Becton, Dickinson and Company, Sparks, USA) were collected at the logarithmic growth phase, washed twice with PBS, and adjusted according to the OD_600_ value. All procedures were approved by the Genetic Recombination Experiment and Pathogen Handling Experiment Committee of Kyorin University (No. 205).

### Mouse wound infection and the administration of phiMR003

The Animal Experiment Committee of Kyorin University (No. 224) approved all procedures involving animal experiments. Female five-week-old BALB/c mice were obtained from CLEA Japan Inc. (Tokyo, Japan) and maintained under specific pathogen-free conditions with free access to food and water in the vivarium at the animal facility at Kyorin University for a week before the experiment. Prior to surgery, mice were deeply anaesthetized by an intraperitoneal (*i.p.*) injection of a cocktail containing medetomidine hydrochloride (0.3 mg/kg), midazolam (4.0 mg/kg), and butorphanol tartrate (5.0 mg/kg), and dorsal hair was shaved and removed with depilatory cream. A full-thickness skin excision wound 6.5 mm in diameter was created with microscissors under a microscope, and a bacterial suspension of 20 µL, including 2 × 10^10^ CFU/mL, was added to the wound site. For phage administration, 20 µL of 1.0 × 10^10^ PFU of phiMR003 in SM buffer without gelatine was added 30 min after MRSA challenge. Wounds were covered with sterile DuraSeal (Diversifle Biotech Inc, Needham, USA) and Tegaderm (3 M, St Paul, USA). Then, the mice were kept in individually ventilated cages after Antisedan *i.p*. injection. All experiments were performed with five or six animals per group.

### Wound closure analysis

Dorsal wound closure was monitored with a digital camera (Finepix F600 EXR, FujiFilm, Tokyo). The wound area was measured using ImageJ software (NIH). The percentage change was calculated for each mouse using 100% as the wound area of each mouse at the start of the experiment.

### Determination of bacterial burden and the quantification of IL-1β, IL-6, and TNF-α

Cytokines were quantified using the following ELISA kits: the IL-1β Quantikine ELISA kit (R&D Systems, Minneapolis, MN), IL-6 Quantikine ELISA kit (R&D Systems, Minneapolis, MN), and TNF-α Quantikine HS ELISA Kit (R&D Systems, Minneapolis, MN). The skin tissue was excised and homogenized in PBS. The number of viable bacterial cells in the tissue was determined by counting the number of colonies formed on mannitol salt medium (Nissui Pharmaceutical, Tokyo, Japan) supplemented with 10 µg/mL ampicillin. Excised tissues were homogenized in lysis buffer 2 (R&D Systems, Minneapolis, MN). After preparation according to the manufacturers’ instructions, lysates were stored at − 80 °C until the quantification of IL-1β. Blood samples were obtained via cardiac puncture under inhalation anaesthesia. After incubation for two hours at room temperature, serum was prepared and stored at − 80 °C until the quantification of IL-6 and TNF-α.

### Myeloperoxidase assay

The MPO activity was analysed as described previously^65^ with some modifications. Excised tissues were homogenized in potassium phosphate buffer (50 mM, pH 6.0) supplemented with hexadecyltrimethylammonium bromide (HTAB). After centrifugation at 13,000 rcf for 15 min at 4 °C, the supernatants were stored at − 80 °C until assay. Ten microlitres of the sample was mixed with 140 µl of assay buffer containing *o*-dianisidine dihydrochloride (D-2679, Sigma‒Aldrich, St. Louis, USA) and 0.05% hydrogen peroxide (Sigma‒Aldrich, St. Louis, USA). The change in absorbance (A_490_) was measured 20 times every 20 s (Multiskan FC, Thermo Fisher Scientific, Waltham, USA). Chromogen was used to generate a standard curve, and data are presented as MPO activity (U/mg tissue weight).

### Immunofluorescence staining and confocal microscopy

Excised tissues were fixed in 4% paraformaldehyde in PBS solution (PFA-PBS) at room temperature for 3 h and treated with 15% sucrose in PBS for 5 h, 30% sucrose in PBS for 5 h, 50% sucrose-OCT for 30 min, and 100% OCT for 15 min. The tissues were embedded in OCT using Histo-Tek PINO-600 (Sakura Finetek Japan Co., Ltd., Tokyo, Japan), and 14 µm frozen sections were prepared using a cryostat (Leicacm 1950, Leica Biosystems, Heidelberg, Germany). Samples were stained with the following primary antibodies: rat anti-Ly-6G (1:200, AdipoGEn Life Science, Liestal, Switzerland, clone Nimp-R14), rat anti-F4/80 (1:200, Abcam, Cambridge, UK, ab6640) and rat anti-ER-TR7 (1:100, BMA Biomedicals, Augst, Switzerland, T-2109) antibodies followed by washing and incubation with host matched secondary antibodies conjugated with AlexaFluor 488 or 594 (1:500, Cell Signaling Technology, Danvers, USA, #4416 and #8889, respectively). Images were acquired by a confocal laser microscope (LSM980, Zeiss, Oberkochen, Germany).

### Isolation of mouse peritoneal macrophages and culture conditions

Three millilitres of 4.05% Brewer's thioglycolate broth was intraperitoneally injected into ten-week-old female C57BL/6 J mice (CLEA Japan, Inc.). Four days later, peritoneal cells were harvested with Dulbecco's modified Eagle’s medium (DMEM). The cells were washed with DMEM and suspended in DMEM supplemented with 10% FBS in 100 unit/mL ampicillin and 100 µg/mL streptomycin at 1.0 × 10^6^ cells/mL, and 1 mL of cell suspension was added to 12-well plates. After 2 h of incubation at 37 °C and 5% CO_2_, nonadherent cells were removed by washing with DMEM, and adherent monolayer cells were treated with phiMR003 (1.0 × 10^8^ PFU/ml, MOI = 100) at 37 °C and 5% CO_2_ for one hour. After the addition of LPS from *Escherichia coli* O26:B6 **(**Sigma‒Aldrich, St. Louis, USA) at a final concentration of 100 ng/ml, macrophages were incubated at 37 °C and 5% CO_2_ for 12 h.

### Isolation of total RNA and analysis by real-time qPCR

The macrophages were washed with PBS, and total RNA was extracted using RNAiso Plus (Takara, Otsu, Japan). cDNA was synthesized using 500 ng of total RNA with an RNA PrimeScript™ 1st Strand cDNA Synthesis Kit (Takara, Otsu, Japan). qPCR was performed using the primers and probes listed in Table 1 with the Probe qPCR Mix (Takara, Otsu, Japan) and the Applied Biosystems 7500 Real-Time PCR System at 95 °C (30 s), followed by 40 cycles at 95 °C (5 s)/60 °C (30 s). To determine the fold changes in the specific mRNAs, transcripts of tested genes were calculated by the ΔΔCt method normalized to the expression of 18S rRNA as an internal control, and the expression levels of IL-1β, IL-6, and TNF-α were compared. For each sample, data were normalized to the expression level of 18S rRNA.

**Table 1:** Primers and probes used for real-time PCR.

Gene	Probe	Forward primer sequence	Reverse primer sequence
Il1b	#78 (Roche Life Science)	5'-TGT AAT GAA AGA CGG CAC ACC-3'	5'-TCT TCT TTG GGT ATT GCT TGG-3'
Il6	#6 (Roche Life Science)	5’-GAT GGA TGC TAC CAA ACT GGA-3’	5’-CCA GGT AGC TAT GGT ACT CCA GAA-3’
Tnfa	#49 (Roche Life Science)	5'-TCT TCT CAT TCC TGC TTG TGG-3'	5'-GGT CTG GGC CAT AGA ACT GA-3’
Rn18s	#55 (Roche Life Science)	5'-AAA TCA GTT ATG GTT CCT TTG GTC-3'	5'-GCT CTA GAA TTA CCA CAG TTA TCC AA-3'

### Statistical analysis

The results are presented as the mean ± standard error of the mean (SEM). Statistical significance was analysed by two-way analysis of variance (ANOVA) or the Mann‒Whitney U test using GraphPad Prism 8.0 (GraphPad Software Inc., San Diego, CA, USA). A statistically significant difference was defined as *P* < 0.05.

## Supplementary Information

Supplementary Information.

## Data Availability

All data generated or analysed during this study are included in this published article and its Supplementary Information files.
