# Supplementary material for: Modification of the immune response by bacteriophages alters methicillin-resistant Staphylococcus aureus infection
Source: Sci Rep. 2022 Sep 19;12:15656. doi: 10.1038/s41598-022-19922-x (PMC9483902; doi:10.1038/s41598-022-19922-x)
Supplement: Supplementary file 1 — Supplementary Information. [file 41598_2022_19922_MOESM1_ESM.pdf]

**Modification of the immune response by bacteriophage alters methicillin resistant *Staphylococcus aureus* infection.** Suda T, Hanawa T, Tanaka M, Tanji Y, Miyanaga K, Hasegawa-Ishii S, Shirato K, Kizaki K, Matsuda T

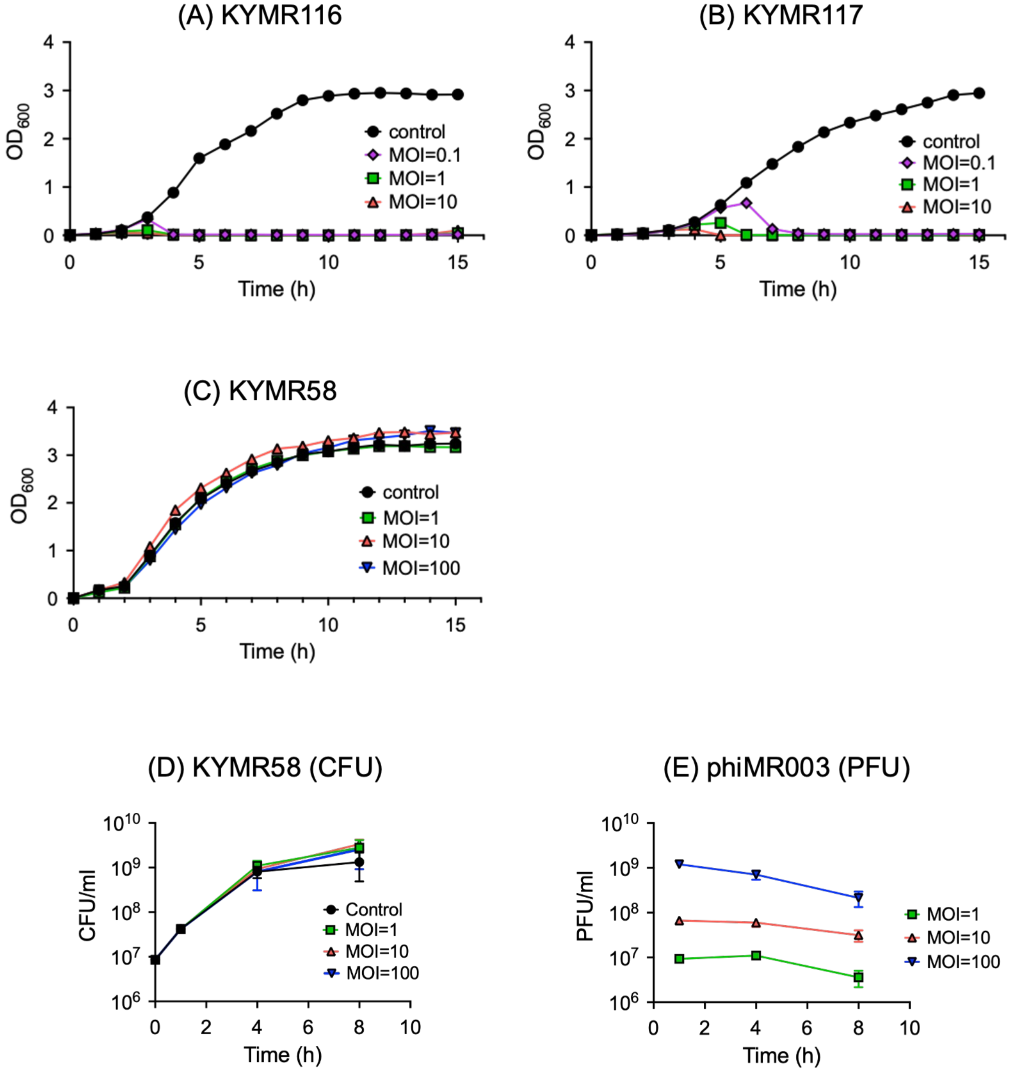


**Supplemental Fig. S1 Sensitivity of MRSA strains to phiMR003.** phiMR003 was added to the culture of KYMR116 (A), KYMR117 (B) and KYMR58 (C) at the MOIs indicated in the figure, and the turbidity ($OD_{600}$) was measured over time. Viable number of bacteria (D) and phiMR003 (E) in the culture of KYMR58 were determined at 1, 4 and 8 hours after the addition of phiMR003 by the plate method and plaque assay, respectively.

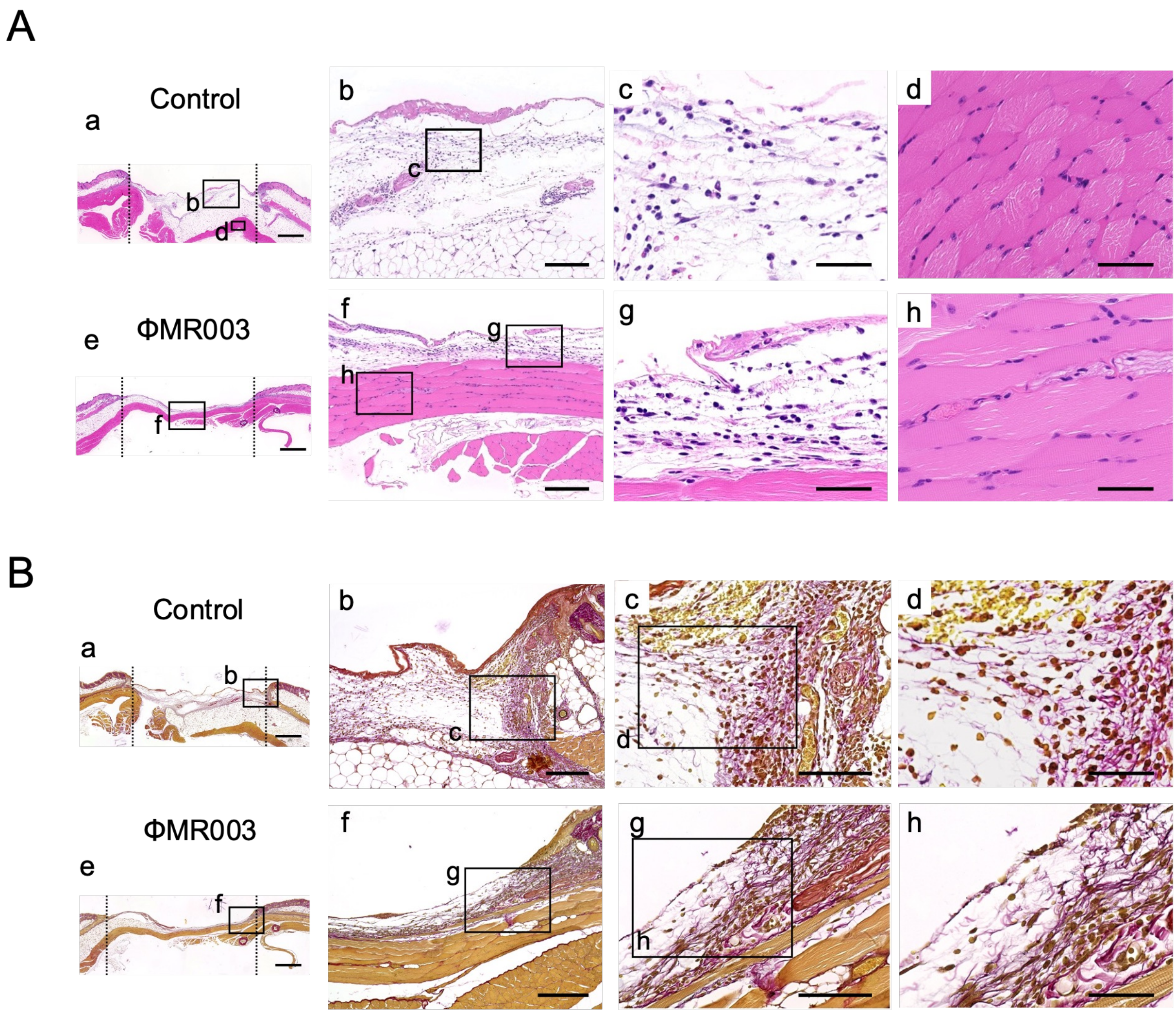


**Supplemental Fig. S2 Micrographs of mouse wound skin tissues.** After excision of dorsal mouse skin, SM buffer (control) or phiMR003 was administered to the wounds. Tissues were fixed with paraformaldehyde at 48 hours after administration and haematoxylin and eosin (A) and Elastica van Gieson (B) stained wound tissues are shown. Micrographs of a-f are the areas

indicating with the squares a-f, respectively. (A). Scale bars, 1 mm in a and e, 200 μm in b and f, and 100 μm in c and g, and 50 μm in d and h in (B).
